# Intranasal Immunization with a Recombinant Avian Paramyxovirus Serotypes 2 Vector-Based Vaccine Induces Protection against H9N2 Avian Influenza in Chicken

**DOI:** 10.3390/v14050918

**Published:** 2022-04-28

**Authors:** Wenhao Yang, Jing Dai, Jingjing Liu, Mengjiao Guo, Xiaowen Liu, Shunlin Hu, Min Gu, Jiao Hu, Zenglei Hu, Ruyi Gao, Kaituo Liu, Yu Chen, Xiufan Liu, Xiaoquan Wang

**Affiliations:** 1 Animal Infectious Disease Laboratory, School of Veterinary Medicine, Yangzhou University, Yangzhou 225000, China; yangwenhao1991@163.com (W.Y.); 15849548893@163.com (J.D.); liujingjing@jit.edu.cn (J.L.); guomj@yzu.edu.cn (M.G.); xwliu@yzu.edu.cn (X.L.); slhu@yzu.edu.cn (S.H.); gumin@yzu.edu.cn (M.G.); hujiaohot@163.com (J.H.); 007622@yzu.edu.cn (R.G.); biochenyu@hotmail.com (Y.C.); 2 Jiangsu Co-Innovation Center for Prevention and Control of Important Animal Infectious Diseases and Zoonosis, Yangzhou University, Yangzhou 225000, China; zengleihu@163.com (Z.H.); liukaituo3@163.com (K.L.); 3 Jiangsu Key Laboratory of Zoonosis, Yangzhou University, Yangzhou 225000, China; 4 Joint International Research Laboratory of Agriculture and Agri-Product Safety of Ministry of Education of China, Yangzhou University, Yangzhou 225000, China

**Keywords:** H9N2, avian paramyxovirus serotype 2, vaccine, intranasal delivery

## Abstract

Commercial inactivated vaccines against H9N2 avian influenza (AI) have been developed in China since 1990s and show excellent immunogenicity with strong HI antibodies. However, currently approved vaccines cannot meet the clinical demand for a live-vectored vaccine. Newcastle disease virus (NDV) vectored vaccines have shown effective protection in chickens against H9N2 virus. However, preexisting NDV antibodies may affect protective efficacy of the vaccine in the field. Here, we explored avian paramyxovirus serotype 2 (APMV-2) as a vector for developing an H9N2 vaccine via intranasal delivery. APMV-2 belongs to the same genus as NDV, distantly related to NDV in the phylogenetic tree, based on the sequences of Fusion (F) and hemagglutinin-neuraminidase (HN) gene, and has low cross-reactivity with anti-NDV antisera. We incorporated hemagglutinin (HA) of H9N2 into the junction of P and M gene in the APMV-2 genome by being flanked with the gene start, gene end, and UTR of each gene of APMV-2-T4 to generate seven recombinant APMV-2 viruses rAPMV-2/HAs, rAPMV-2-NPUTR-HA, rAPMV-2-PUTR-HA, rAPMV-2-FUTR-HA, rAPMV-2-HNUTR-HA, rAPMV-2-LUTR-HA, and rAPMV-2-MUTR-HA, expressing HA. The rAPMV-2/HAs displayed similar pathogenicity compared with the parental APMV-2-T4 virus and expressed HA protein in infected CEF cells. The NP-UTR facilitated the expression and secretion of HA protein in cells infected with rAPMV-2-NPUTR-HA. Animal studies demonstrated that immunization with rAPMV-2-NPUTR-HA elicited effective H9N2-specific antibody (6.14 ± 1.2 log2) responses and conferred complete immune protection to prevent viral shedding in the oropharyngeal and cloacal swabs from chickens challenged with H9N2 virus. This study suggests that our recombinant APMV-2 virus is safe and immunogenic and can be a useful tool in the combat of H9N2 outbreaks in chicken.

## 1. Introduction

H9N2 avian influenza virus (AIV), initially isolated from turkeys in America in 1966, is known as low pathogenic avian influenza virus. In the following decades, H9N2 was endemic in farmed poultry in most parts of Asia, the Middle East and North Africa, and West Africa [1]. Not only does it cause severe economic losses to the poultry industry [2,3], but it may cause cross-species transmission, posing a threat to public health [4,5,6]. Study shows that seroprevalence rate of antibodies to H9N2 among poultry workers reached 11.20% in several provinces of China during 2014–2016 [7]. Almost all H9N2 AIVs have acquired human-type receptor-binding abilities [8]. Therefore, it is crucial to control the spread of H9N2 virus in chicken flocks, whether it is for the development of the poultry industry or human health.

For a long time, immunization with inactivated vaccine was the main method to prevent AIV in chicken [9,10]. In 1998, the first inactivated vaccine was issued to control H9N2 in chicken in China [11]. Although inactivated vaccines can induce humoral immune responses, they induce weak cellular immune responses and only can provide limited immunogenicity [12]. Additionally, H9N2 has become the dominant AIV subtype in both chickens and ducks during 2016–2019 [8], which suggests that there is not enough evidence to prove that the inactivated vaccine is effective in controlling the spread of H9N2 [13].

Live vector-based vaccines typically provide humoral, cellular, and mucosal immunity, compare with inactivated vaccines [14,15,16,17]. The vector vaccine expressing HA protein of H9N2 virus is an ideal substitute for inactivated vaccine [18,19]. There are some viral vectors commonly used to construct live vaccines of avian recombinant viruses, such as fowl pox virus [20], Newcastle disease virus [21], adenovirus [22], Marek’s disease virus [23], and Herpesvirus of Turkeys [24]. The NDV vector vaccine expressing HA protein could be administered by drinking water, and induce mucosal antibody and cellular immunity [25], which can protect poultry from AIV infection [26]. However, the main problem of the recombinant vaccine is whether the vaccinated birds have immunity to the vector, or whether the host range of the vector virus is limited [13]. For NDV, most commercial poultries are immunized with NDV vaccine and young birds will have maternal anti-NDV antibodies [10,18,27,28]. Avian paramyxovirus serotype 2 (APMV-2) belongs to the same genus as NDV and infects a wide variety of avian species, but does not cause any apparent disease in chickens [29]. The serum antibodies inducing by APMV-2 were very low cross-reacted significantly with NDV [30,31,32,33]. Therefore, it can circumvent maternal-derived anti-NDV antibodies. APMV-2 vector vaccine expressing HA protein of H9N2 can be used as an emergency vaccination of chickens [33]. In addition, whether the insertion of the HA gene has any effect on the pathogenicity of APMV-2 is also a matter of concern. Recombinant vector vaccines must be safe for targeted animals. Previous study has shown that the insertion of foreign HA genes did not increase the pathogenicity of APMV-2 to chickens. APMV-2 recombinant vaccine expressing H5-HA gene could provide certain protection against HPAIV [32].

The genome of APMV-2 contains six open reading frames (ORFs), including nucleoprotein (NP), phosphoprotein (P), matrix protein (M), fusion glycoprotein (F), hemagglutinin-neuraminidase (HN), and polymerase protein (L). Each ORF contains gene start (GS) sequence and gene end (GE) sequence, with untranslated regions (UTRs) between the ORF and GS or GE. In this study, we performed whole-genome sequencing of APMV-2-T4, and successfully established a reverse genetics rescue system. Previous research demonstrated that the insertion of flanking UTRs of APMV-1 NDV could affect the expression of an exogenous gene [34]. APMV-10 UTRs could enhance the expression of the exogenous HA gene, improving the vaccine efficacy of rAPMV-10/HA in chickens, especially the 5′ and 3′ UTRs of NP gene [35]. Therefore, we generated seven recombinant APMV-2-UTR-HAs(rAPMV-2/HAs) expressing H9N2 HA gene flanked by 5′ and 3′ UTR of each gene, and evaluated their vaccine efficacy in chickens.

## 2. Materials and Methods

### 2.1. Viruses and Cells

The APMV-2-T4 strain was kindly given by Prof. Jixun Zhao (China Agricultural University). The H9N2 virus strain A/chicken/Anhui/AH463/2017 (AH463) was obtained from the Animal Infectious Disease Laboratory, School of Veterinary Medicine, and Yangzhou University, and specify biosafety level 2 (BSL-2) used for work with it. The H9N2 virus was grown in 9-day-old specific pathogen free (SPF) embryonated chicken eggs. The SPF embryonated chicken eggs were supplied from Zhejiang Lihua Agricultural Technology Co., Ltd. (Zhejiang Lihua Agricultural Technology Co., Ltd., Zhejiang, China). BHK-21 cells, clone BSR T7/5, stably expressing the phage T7 RNA polymerase, developed by Buchholz et al. [36], were a gift from Prof. Zhigao Bu (Harbin Veterinary Institute, China). Primary chicken embryonic fibroblasts (CEF) and BSR T7/5 were grown and maintained in Dulbecco’s Modified Eagle’s Medium supplemented with 10% fetal bovine serum respectively, at 37 °C with 5% CO_2_ atmosphere.

### 2.2. Whole Genome Sequencing of APMV-2-T4

Based on the genomic sequences of APMV-2 strains available in GenBank, 10 pairs of primers were designed to obtain the complete genome information of APMV-2-T4 isolate. Primer Premier 5.0 was used to design the primers (listed in Table S1). The overlap sequence between adjacent segments was at least 40 bp. Ten segments of APMV-2-T4 were amplified through reverse transcriptase PCR. 5′ and 3′ *RACE* of APMV-2-T4 was performed using the SMARTer 5′/3′ RACE Kit (Takara Bio, Dalian, China) according to the manufacturer’s instructions. After sequencing, the whole genome sequence was spliced by using SEQMAN in the lasergene package. MEGA 5.0 software was used to construct the phylogenetic trees of the genome sequence of APMV-2-T4 and other *Paramyxoviridae* representative strains. Reference sequences were selected from the GenBank database, as shown in Table S2.

### 2.3. Construction of Plasmids

The full-length cDNA of APMV-2-T4 was assembled into the transcription plasmid TVT by using pEASY^®^-Basic Seamless Cloning and Assembly Kit (Transgen, Beijing, China), named TVT-T4. The NP, P, and L genes of APMV-2-T4 were cloned into the pCI vector to construct derived helper expression plasmids, named pCI-NP, pCI-P, and pCI-L. The HA gene of H9N2 (GenBank no. ON248014.1) was used as the exogenous antigen gene, which was amplified with HA-F and HA-R (Table S3). The UTR sequences flanked with homology sequence of HA and vector were generated by annealed oligo primers, shown as Table S3. Then HA gene flanked with the GS, GE, and UTR sequences of each gene of APMV-2-T4 was amplified by overlap PCR. The overlap PCR products were inserted into the P and M gene junction of APMV-2-T4 by using pEASY^®^-Basic Seamless Cloning and Assembly Kit. The plasmids of TVT-T4/HAs (TVT-T4-NPUTR-HA, TVT-T4-PUTR-HA, TVT-T4-FUTR-HA, TVT-T4-HNUTR-HA, TVT-T4-LUTR-HA, TVT-T4-MUTR-HA, and TVT-T4-nonUTR-HA) were constructed.

### 2.4. Rescue of the Viruses

The rAPMV-2/HAs viruses were generated by reverse genetics, as described previously [21]. BSR T7/5 cells were seeded at a density of 0.6 × 10^6^ cells/well in six-well plates. Briefly, TVT-T4/HAs or TVT-T4, pCI-NP, pCI-P, and pCI-L were transfected into BSR T7/5 cells using X-tremeGENE HP DNA Transfection Reagent (Roche, Mannheim, Germany), respectively. After 96 h post-transfection, the transfected culture supernatants and cell monolayers were harvested and inoculated into the allantoic cavity of 9-day-old embryonated chicken eggs for virus recovery. After 96 h inoculation, allantoic fluid was collected, and the rescued viruses were confirmed by hemagglutination (HA) test with chicken red blood cells. The rAPMV-2/HAs were verified by primers T4-HA-F and T4-HA-R (Table S3).

### 2.5. Evaluation of Biological Characteristics of Recombinant Viruses rAPMV-2/HAs

Titers of rescued viruses were detected by HA test. The 50% egg infection dose (EID_50_) virus was performed by inoculating serial 10-fold dilutions of the recombinant viruses or parental virus into 9-day-old embryonated chicken eggs. The Median Tissue Culture Infectious Dose (TCID_50_) test was performed by inoculating serial 10-fold dilutions of the chimeras or parental virus into CEF, as described by Alexander [37]. The values of EID_50_ and TCID_50_ were calculated by the Reed and Muench method [38]. To characterize the pathogenicity of viruses, mean death time (MDT) was determined by 9-day-embryonated chicken eggs [37]. Intracerebral pathogenicity index (ICPI) in one-day-old SPF chicks were performed according to the recommendations of the World Organization for Animal Health (OIE) [37,39].

### 2.6. Determination of HA Protein Expression

CEF were infected with rAPMV-2/HAs at a multiplicity of infection (MOI) of 1. APMV-2-T4 and H9N2 were used as negative and positive control respectively. The expression of HA protein was evaluated by western blot analysis using polyclonal chicken anti-H9N2 serum (prepared by our lab), and Goat anti-Chicken IgY (H + L) Secondary Antibody, HRP (Thermo Fisher Scientific, Waltham, MA, USA). β-actin (monoclonal anti-β-actin; Sigma Chemical, St. Louis, MO, USA) was used as an internal control.

### 2.7. Vaccination Efficacy in Chickens

Five-week-old SPF white Leghorn chickens were purchased from JINAN SPAFAS POULTRY Co., Ltd. (Jinan, China), and were randomly divided into seven groups. Seven groups of chicken were vaccinated by the intranasal and intraocular route with 10^6^ EID_50_ of rAPMV-2-NPUTR-HA, rAPMV-2-PUTR-HA, rAPMV-2-FUTR-HA, rAPMV-2-HNUTR-HA, rAPMV-2-LUTR-HA, APMV-2-T4, and phosphate buffered saline (PBS) in a 0.1 mL volume, respectively. Chickens in the control group were inoculated the same way with PBS. Blood samples were collected weekly from each bird in each group until 4 weeks post-vaccination (wpv). Serum antibody titers against H9N2 were measured using hemagglutination inhibition (HI) test. At 4 wpv, chickens were challenged through intravenous injection with H9N2 at 0.1 mL (10^6^ EID_50_). At 3, 5, and 7 days post challenge (dpc), oropharyngeal (O) and cloacal (C) swabs were collected from all the chickens for virus shedding detection using SPF embryonated chicken eggs (3 eggs per swab).

### 2.8. Statistical Analyses

Statistical analyses were conducted by Student’s *t*-test using GraphPad Prism 8.0 (GraphPad Software, La Jolla, CA, USA). Differences were considered statistically significant at *p* values of < 0.05.

## 3. Results

### 3.1. Phylogenetic and Genetic Analysis of APMV-2-T4

**Figure 1:**
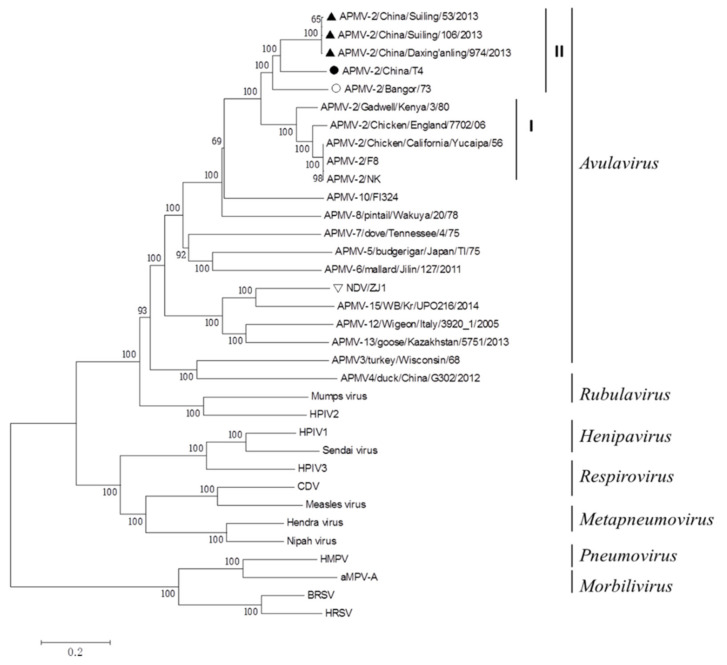
Phylogenetic tree of representative members of the family *Paramyxoviridae* based on the complete genomes. A neighbor-joining tree was generated using MEGA 5.0, and a 1000-bootstrap analysis was performed. The sequence of APMV-2-T4 is marked with a solid black circle. The sequence of others APMV-2 isolated from China are marked with Solid Upright Triangle. The sequence of APMV-2 isolated from Bangor is marked with hollow black circle. The representative strain of NDV VIId ZJ1 is marked with hollow inverted triangle. Others *Paramyxoviridae* viruses were found on the NCBI and the GenBank numbers are shown in Table S2.

The genome sequence of APMV-2-T4 consists of 15,000 bp. A phylogenetic tree was generated based on the complete genomes of family *Paramyxoviridae*. APMV-2-T4 remained in the same cluster with three strains of APMV-2 isolated from China, and the sequences homology is 77.4–77.6%. APMV-2/Suiling/106/2013 has the highest homology with APMV-2-T4 (77.6%). The foreign representative strain with the highest homology with APMV-2-T4 strain is APMV-2/Bangor/73, with a homology of 77.2% (Figure 1). These five viruses were placed in genetic group II.

The F gene sequence of APMV-2-T4 consisted of 1707 bp and its ORF is 1611 bp. The amino acid sequence of F protein of APMV-2-T4 was compared with China/Suiling/106/2013, APMV-2/Bangor/73, APMV-2/Chicken/Califormia/Yucaipa/56, and ZJ1 which was the representative strain of NDV VIId. The sequence of F protein of APMV-2-T4 has 69.5–78.3% homology with that of F protein of other APMV-2. However, the homology between APMV-2-T4 strain and ZJ1 is only 49.6%. The HN gene sequence of APMV-2-T4 consisted of 1899 bp and its ORF is 1743 bp. Similar to the F protein, the homology of the HN protein between APMV-2-T4 and other APMV-2 is 67.5–78.9%. The homology between APMV-2-T4 strain and ZJ1 is only 44.3% (Table 1).

### 3.2. Generation of rAPMV-2/HAs Expressing HA Protein of H9N2

**Figure 2:**
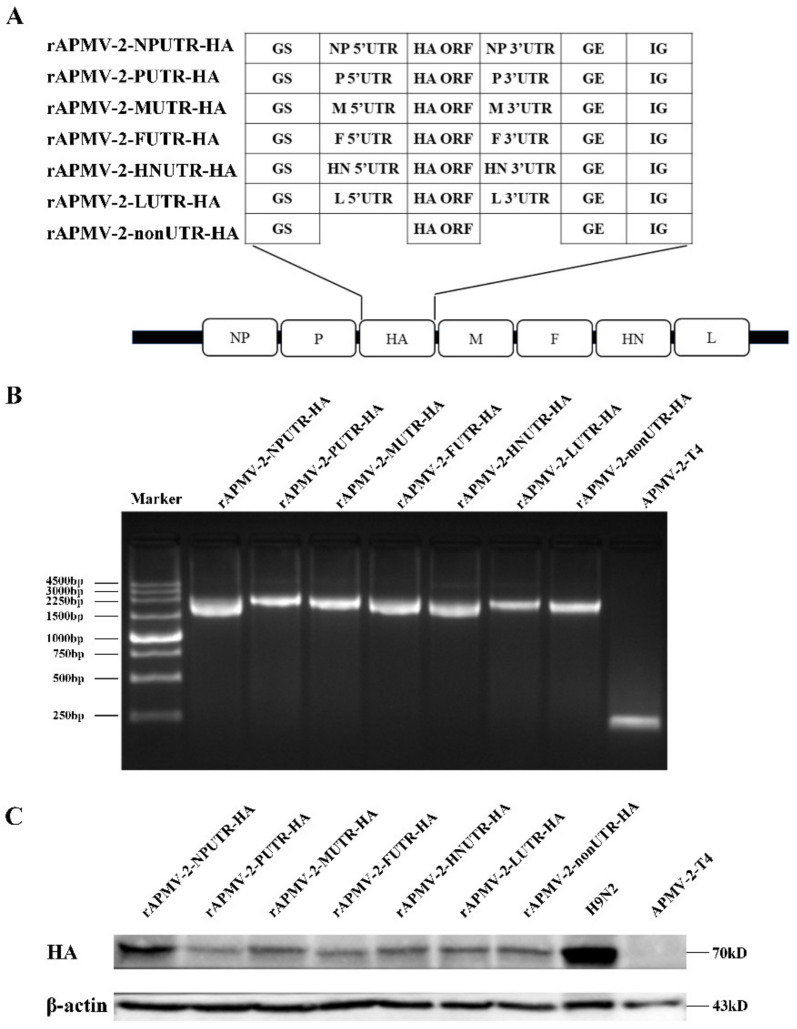
Construction of recombinant avian paramyxovirus serotype 2 (APMV-2) expressing HA gene of H9N2. (**A**) The schematic diagram for the construction of rAPMV-2 expressing HA gene of H9N2. The upstream flanking of the HA gene is the gene start sequences and 5′ UTRs for each APMV-2-T4 gene, and the downstream flanking is 3′ UTRs for each APMV-2-T4 gene and the gene end sequences. rAPMV-2-nonUTR do not contain any UTRs, only the gene start sequence and gene end sequence. All chimeric structures were inserted between the P and M genes in the APMV-2-T4 genome. (**B**) The allantoic fluid of rAPMV-2/HAs and APMV-2-T4 were collected, respectively, for PCR analysis. (**C**) Expression level of the HA proteins in rAPMV-2/HAs were confirmed by western blot analysis in infected CEF. Equal protein loading was confirmed with the β-actin antibody.

The reverse genetics system of APMV-2-T4 was constructed, include of full-length cDNA clone of APMV-2-T4 and three derived helper expression plasmids. The HA gene, flanked by UTRs of each gene of APMV-2-T4, was inserted between P and M gene of APMV-2-T4 (Figure 2A). rAPMV-2-NPUTR-HA, rAPMV-2-PUTR-HA, rAPMV-2-FUTR-HA, rAPMV-2-HNUTR-HA, rAPMV-2-LUTR-HA, rAPMV-2-MUTR-HA, and rAPMV-2-nonUTR-HA were successfully constructed. Insertion of chimeric HA gene was confirmed by PCR amplification with site-specific primers (Figure 2B). To confirm the expression of HA protein of rAPMV-2/HAs, total cell lysates from infected CEF were analyzed by western blot (Figure 2C). CEF infected with rAPMV-2/HAs showed approximately a ~70 kDa of HA protein, the same as the positive control sample infected with H9N2, while no HA band was observed in CEF lysates infected with APMV-2-T4. The expression of HA protein was highest in cells infected with rAPMV-2-NPUTR-HA. In addition, no differences in the expression of HA protein were observed when cell lysates infected with rAPMV-2/HAs were analyzed after 15 passages (Figure S1).

### 3.3. The Virus Titre and Pathogenicity Indexes of rAPMV-2/HAs

In order to measure the titers of rAPMV-2/HAs, a standard HA titer assay was conducted. The HA titers of rAPMV-2-NPUTR-HA, rAPMV-2-PUTR-HA, rAPMV-2-FUTR-HA, rAPMV-2-HNUTR-HA, and rAPMV-2-LUTR-HA were 8 or 9 log_2_, which was slightly higher than that of the APMV-2-T4 strain. However, the HA titers of rAPMV-2-MUTR-HA and rAPMV-2-nonUTR-HA were only 5 log_2_. The EID_50_ of the rescued virus were the same trend as the HA titers. Therefore, rAPMV-2-MUTR-HA and rAPMV-2-nonUTR-HA were discarded in the next immune experiment. The MDT observed for all rAPMV-2/HAs were greater than 120 h. The results of ICPI for all rAPMV-2/HAs were less than 0.7 (Table 2).

### 3.4. HI Antibody Titers of rAPMV-2/HAs against H9N2 or APMV-2

**Figure 3:**
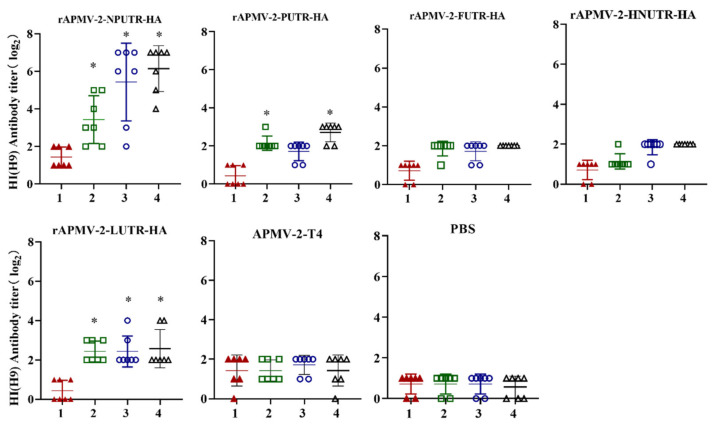
HI (H9) antibody titers induced by rAPMV-2/HAs. Five-week-old chickens were vaccinated with 10^6^ EID_50_ rAPMV-2-NPUTR-HA, rAPMV-2-PUTR-HA, rAPMV-2-FUTR-HA, rAPMV-2-HNUTR-HA, rAPMV-2-LUTR-HA, APMV-2-T4, and PBS, respectively. The serum was collected at 1, 2, 3, and 4 weeks post-vaccination. HI antibody titers against H9N2 virus were detected (n = 7). Axis X—weeks post-vaccination. Statistical analyses were performed by Student’s *t*-test between rAPMV-2/HAs vaccine groups and PBS group at the same time points. * *p* < 0.05.

To assess the immunogenicity induced by rAPMV-2/HAs, sera were collected weekly from chickens immunized with rAPMV-2-NPUTR-HA, rAPMV-2-PUTR-HA, rAPMV-2-FUTR-HA, rAPMV-2-HNUTR-HA, rAPMV-2-LUTR-HA, APMV-2-T4, and PBS via the intranasal and intraocular, respectively. Our results showed that the HI antibody titers induced by rAPMV-2-NPUTR-HA were highest; the earliest immune responses against H9N2 were at 2 wpv. At 4 wpv, the HI antibody titers induced by rAPMV-2-NPUTR-HA reached 6.14 ± 1.2 log_2_. In addition, 5/7 chickens vaccinated rAPMV-2-NPUTR-HA had more than 6 log_2_ HI antibody titers. However, the HI antibody titers induced by others rAPMV-2/HAs no more than 3 log_2_ (Figure 3). These results showed that rAPMV-2-NPUTR-HA was highly immunogenic to induce humoral immunity.

### 3.5. Protective Efficacy of Recombinant APMVs against H9N2 AIV

No clinical symptoms were observed in chickens in the vaccine group and PBS group after challenged with H9N2 virus. The H9N2 virus belongs to low pathogenic AIV, the virus shedding was a commonly used criterion to evaluate the protective efficiency of the rAPMV-2/HAs against H9N2 virus. The oropharyngeal and cloacal swab samples were collected for at 3, 5, and 7 dpc. All chickens (7/7) in APMV-2-T4 or PBS group shed virus after challenged with H9N2 virus through intravenous injection at 3 dpc. However, no virus was detected in oropharyngeal and cloacal swab samples in chickens vaccinated with rAPMV-2-NPUTR after challenged with H9N2 virus through intravenous injection at 3, 5, and 7 dpc. In addition, only 2 chickens (2/7) in rAPMV-2-LUTR group shed virus at 3 and 5 dpc, no virus was detected at 7 dpc. Immunization with rAPMV-2-PUTR, rAPMV-2-FUTR, and rAPMV-2-HNUTR slightly inhibited the virus shedding (Table 3). These results demonstrated that virus shedding in chickens immunized with rAPMV-2-NPUTR were completely inhibited after challenged with H9N2 virus through intravenous injection.

## 4. Discussion

H9N2 AIV has been wildly spread and become the more devastating subtype of AIV for poultry industry in China [40]. Since 1998, China has used inactivated vaccines to prevent chickens from the H9N2 virus infection. Inactivated vaccines have defects such as multiple use, must be injected intramuscularly, and require more labor. The stress response caused by multiple intramuscular injections may cause harm, such as slow growth and decreased egg production [26]. In addition, the isolation rate of H9N2 virus from chickens has ranked first among all subtype AIV in China [8], which suggests that the H9N2 inactivated vaccine was not effective enough. There is an urgent need for a novel live vaccine to control the H9N2 virus. In this study, the reverse genetics system of APMV-2-T4 was constructed to expression HA of H9N2 virus. Serum antibodies against APMV-2 have low cross-reactivity with NDV [41]. The rAPMV-2/HAs vaccine can avoid the interference of immune effect by maternal antibodies of NDV. Seven recombinant rAPMV-2/HAs expressing HA gene of H9N2 were developed through a reverse genetic system.

APMV can infect a wide range of poultry worldwide, with different clinical symptoms and economic impact, and 20 serological types (1–20) of APMV have been isolated. Through systematic genomics analysis, all APMVs are clustered into three different clades, and APMV-2 belongs to the major clade-I [42]. According to the reported classification method of APMV-2 strains [43], the APMV-2-T4 strain, three strains previously isolated in China, and APMV-2/Bangor/73 jointly constitute the gene type II. However, APMV-2-T4 was quite different from the known APMV-2 strain in genome sequence homology, the sequences homology is only 77.4–77.6%. The genome sequence of APMV-2-T4 shared the highest nucleotide identity with rubulaviruses and the lowest with morbillivirus in the family *Paramyxovirinae*. The genome size of APMV-2-T4 is 15,000 bp, which is consistent with the ‘rule of six’, with the necessary conditions for virus replication and survival. The F and HN proteins are the main protective antigens of avian paramyxovirus. A comparison of amino acid sequence of the F and HN proteins revealed that APMV-2-T4 is distantly related (49.6% and 44.3%, respectively) to NDV, indicating that there is a large antigenic difference. This result further demonstrated that the APMV-T4 strain has the potential as a vaccine vector to resist NDV antibodies. In addition, Ryota Tsunekuni et al. found that the cross-reactivity of APMV-2 with NDV serum was low by HI assay and even was below the minimum detection line by virus neutralization (VN) assay. In NDV-challenged chickens, specific antibodies induced by APMV-2 were unaffected, as was replication of APMV-2. Furthermore, no clinical signs were present in the chickens challenged with APMV-2 [33]. Study has shown that between 2003 to 2005, the seroprevalence of APMV-2 was as high as 42.9% in chicken by HI analysis in China [44]. However, in a recent study, 3144 chicken swab samples were collected from 11 provinces in China and no positive samples of APMV-2 were found by RT-PCR analysis [45], suggesting that the prevalence of APMV-2 is extremely low in flocks and does not affect the application of APMV-2 vector vaccine in field conditions.

APMV-2 has a unique genetic structure “GS-5′ UTR-ORF-3′ UTR-GE”. The UTRs sequence of virus has been confirmed to play an important role in the transcription and translation process of family *Paramyxoviridae*. In canine distemper virus, the virus with deleted 3′ UTR of M gene replicated more efficiently, which is related to the reduction of virulence [46]. The long 5′ UTR of F mRNA and the long 3′ UTR of M mRNA regulated the replication and cytopathogenicity of measles virus by regulating the production of M and F proteins, respectively [47]. UTRs of different gene also plays an important role in the construction of paramyxovirus vaccine vectors expressing foreign genes. In the case of NDV, previous study has constructed recombinant NDVs expressing foreign genes flanked by 3′ UTR and 5′ UTR of each gene of NDV. UTR of M or F could increase the level of mRNA transcription and protein expression of foreign gene, while HN or L played an inhibitory role, and almost no foreign gene expression was detected at the protein level [34]. On the contrary, for APMV-10, UTRs of all six genes could increase the expression of exogenous HA protein [35]. In this study, we demonstrated that UTR of NP gene produced the highest level of HA protein expression in APMV-2, which was different with APMV-1 (NDV) and APMV-10. The mechanism of UTRs regulating protein expression needs to be further studied. The level of protein expression and HA titers of APMV-2-MUTR-HA and APMV-2-nonUTR-HA were low. It was possible that these two modes of constructing recombinant viruses affected the replication efficiency of the virus in chicken embryos. Based on these results, APMV-2-MUTR-HA and APMV-2-nonUTR-HA were not suitable as vaccine candidates, so the two strains did not be tested for immunity experiment. According to the OIE, a definitive assessment of virus virulence is based on the ICPI [48], all rescued recombinant viruses were low virulent strains (ICPI < 0.7), and the insertion of HA gene did not change the virulence.

Serum antibody titers is an important criterion for evaluating the immune protection effect of vaccine. In the present study, only rAPMV-2-NPUTR-HA induced high level of HI antibody titers against H9N2 virus, while HI antibody titers induced by others rAPMV-2/HAs no more than 3 log_2_. This result was consistent with the trend of HA protein expression in CEF cells infected rAPMV-2/HAs. The result demonstrated that only UTR of NP gene was able to effectively augment HA protein expression in APMV-2. The number of birds shedding virus from the oropharynx or cloaca is an important indicator for evaluating efficacy challenge test of LPAI vaccine [49]. Embryonated chicken eggs and real time-PCR were usually used to detect the virus shedding [41,50,51,52]. However, embryonated chicken eggs can detect live virus, but real time-PCR cannot. H9N2 virus adapts well to be cultured in the embryo, and usually had the HA titer by the first isolation in the embryo. In this study, rAPMV-2-PUTR-HA, rAPMV-2-FUTR-HA, rAPMV-2-HNUTR-HA, and rAPMV-2-LUTR-HA induced reduction of viral shedding in chicken challenged H9N2 virus. Specifically, viral shedding from both trachea and cloacae, with challenged H9N2 virus, was not detected in chickens vaccinated with rAPMV-2-NPUTR-HA. The rAPMV-2-NPUTR-HA recombinant virus could induce the differentiation of CD4^+^ and CD8^+^ T cells in chickens (data not published). However, the specific mechanism is still unclear, and the H9 HA-specific cellular immunity and mucosal immune IgA of rAPMV-2-NPUTR-HA recombinant virus will be further explored. It is also important to note that early infection of H9N2 in broilers is a major troubling problem in current field situation. In further study, the protective effect of the vaccine in young chickens (in the first and second weeks of age) and challenge within 1–2 weeks after immunization should be explored.

## 5. Conclusions

In summary, we constructed the reverse genetics system of APMV-2-T4, and seven recombinant rAPMV-2/HA vaccines were successfully rescued. Except for APMV-2-MUTR-HA and APMV-2-nonUTR-HA, which were not tested for immunity experiment due to the low titers of HA and EID_50_. Compared with the UTR sequences of other genes of APMV-2, only the UTR of NP gene could improve the expression of exogenous proteins. All APMV-2/HAs could inhibited the shedding of H9N2 virus. However, only rAPMV-2-NPUTR-HA could induce a high level of HI antibody titers against H9N2 virus. Virus shedding in oropharyngeal and cloacal swabs were completely inhibited in chickens vaccinated with rAPMV-2-NPUTR-HA after challenged with H9N2 virus. Overall, rAPMV-2-NPUTR-HA might be a good candidate vaccine for mass-vaccination of commercial chickens in field conditions.

## Figures and Tables

**Table 1:** Comparison of amino acid identity between APMV-2-T4 strains and NDV/ZJ1 for F and HN proteins.

Strains	F ^a^					HN ^b^				
	ZJ1	T4	Suiling106	Bangor	Yuc	ZJ1	T4	Suiling106	Bangor	Yuc
NDV/ZJ1		49.6	49.4	47.4	46.9		44.3	43.2	42.8	44.8
APMV-2-T4			78.3	71.0	69.5			78.9	71.1	67.5
APMV-2/Suiling106				71.6	69.0				71.5	68.4
APMV-2/Bangor					68.3					64.8
APMV-2/Yucaipa										

^a^ fusion protein; ^b^ hemagglutinin-neuraminidase.

**Table 2:** Biological characteristics of rAPMV-2/HAs and APMV-2-T4.

	HA ^1^ (log_2_)	EID_50_ ^2^ (/0.1 mL)	MDT ^3^ (Hours)	ICPI ^4^
rAPMV-2-NPUTR-HA	8	10^8.63^	>120	0
rAPMV-2-PUTR-HA	8	10^8.33^	>120	0
rAPMV-2-MUTR-HA	5	10^5.33^	>120	0
rAPMV-2-FUTR-HA	8	10^8.17^	>120	0
rAPMV-2-HNUTR-HA	9	10^8.5^	>120	0
rAPMV-2-LUTR-HA	8	10^8.17^	>120	0
rAPMV-2-nonUTR-HA	5	10^5.17^	>120	0
APMV-2-T4	7	10^7.50^	>120	0

^1^ Hemagglutination test; ^2^ 50% egg infection dose; ^3^ mean death time; ^4^ intracerebral pathogenicity index.

**Table 3:** Virus shedding of chickens challenged with H9N2 virus.

	3 dpc		5 dpc		7 dpc	
	O	C	O	C	O	C
rAPMV-2-NPUTR-HA	0/7	0/7	0/7	0/7	0/7	0/7
rAPMV-2-PUTR-HA	4/7	0/7	3/7	0/7	0/7	0/7
rAPMV-2-FUTR-HA	5/7	0/7	3/7	1/7	0/7	0/7
rAPMV-2-HNUTR-HA	5/7	0/7	4/7	0/7	0/7	0/7
rAPMV-2-LUTR-HA	2/7	0/7	2/7	1/7	0/7	0/7
APMV-2-T4	7/7	1/7	4/7	0/7	1/7	0/7
PBS	7/7	1/7	5/7	1/7	2/7	0/7

Five-week-old chickens were vaccinated with 10^6^ EID_50_ rAPMV-2-NPUTR-HA, rAPMV-2-PUTR-HA, rAPMV-2-FUTR-HA, rAPMV-2-HNUTR-HA, rAPMV-2-LUTR-HA, APMV-2-T4, and PBS respectively. Chickens were challenged through intravenous injection with 10^6^ EID50 of H9N2 virus at 4 wpv. Titers of H9N2 virus shedding in oropharyngeal (O) and cloacal (C) swabs on 3, 5, and 7 days post challenge (dpc) were determined using SPF embryonated chicken eggs (3 eggs per swab).

## Data Availability

Not applicable.

## Supplementary Materials

The following supporting information can be downloaded at: https://www.mdpi.com/article/10.3390/v14050918/s1, Table S1: Primers designed for whole genome sequencing of APMV-2/T4; Table S2: The reference strains used in Phylogenetic trees; Table S3: List of primers used for vector plasmid construction and recombinant virus; Figure S1: Stability analysis of APMV-2/HAs.
