# Supplementary material for: Intranasal Immunization with a Recombinant Avian Paramyxovirus Serotypes 2 Vector-Based Vaccine Induces Protection against H9N2 Avian Influenza in Chicken
Source: Viruses. 2022 Apr 28;14(5):918. doi: 10.3390/v14050918 (PMC9144924; doi:10.3390/v14050918)
Supplement: Supplementary file 1 [file viruses-14-00918-s001.zip › viruses-1658599-supplementary.pdf]

Table S1 Primers designed for whole genome sequencing of APMV-2/T4

Primer	Primer sequence	Expected size (bp)
C1F	AACTGACTTCGTGGGGG	1806
C1R	GCTGGGCTGTGCTTTCTC	
C2F	GGGCGAATCCCACTCAAGT	1554
C2R	GCCACCCTTATTTGCTGTTG	
C3F	TGTAGGGCCCTAGACAAACAAGC	1631
C3R	AGGGTGGCCTGGGC	
C4F	GGCAACCAGACATGATACCC	1840
C4R	TCCCCCTATCTCCCTGGC	
C5F	CCACAGTGCACCAAGG	1674
C5R	TCCACGGGTCTTTCCGA	
C6F	CAGGAGTTGCCATCTAAAGAC	656
C6R	CAATTTCCACAATAGCACTAATTTTG	
C7F	AGAATTCATGGACCAAGTCCAAGCG	1872
C7R	GCTGGCTCGCCTCTG	
C8F	CAGAGGCGAGCCAGCAAG	2245
C8R	CTGCGTATGCATCGGGTG	
C9F	CCGATGCATACGCAGTGGATA	2644
C9R	GACTCAAGCACGTTTAAATTTTAGACAAC	

Table S2. The reference strains used in Phylogenetic trees

Virus strain	Accession number	Virus strain	Accession number
NDV/ZJ1	AF431744.3	APMV-13 goose/Kazakhstan/2013	KU646513.1
APMV-2/Chicken/Califormia/Yucaipa/56	EU338414.1	APMV-15/WB/UPO216/2014	KY511044.1
APMV-2/ China/Daxing'anling/974	KT071757.1	CDV	MF041963.1
APMV-2/China/Suiling/53/2013	KT071755.1	HPIV3	KF530250.1
APMV-2/ China/Suiling/106/2013	KT071756.1	Measles virus	KJ410048.1
APMV-2/F8	HQ896023.1	Mumps virus	MF965196.1
APMV-2/NK	HQ896024.1	HPIV2	NC_003443.1
APMV-2/Chicken/England/7702/06	HM159993.1	HPIV1	KF530217.1
APMV-2/Gadwell/Kenya/3/80	HM159994.1	Sendai virus	KT120023.1
APMV-2/Bangor/73	HM159995.1	Hendra virus	AF017149.3
APMV-3/turkey/Wisconsin/68	EU782025.1	Nipah virus	JN808857.1
APMV-4/duck/China/G302/2012	KC439346.1	HMPV	KJ627377.1
APMV-5/budgerigar/Japan/T	LC168750.1	aMPV-A	MF093139.1
APMV-6/mallard/Jilin/127/2011	HM159994.1	BRSV	AF295543.1
APMV-7/dove/Tennessee/4/75	FJ231524.1	HRSV	MF614946.1
APMV-8/pintail/Wakuya/20/78	FJ215864.1		
APMV-10-FI324	LC187309.1		
APMV-12/Wigeon/Italy/2005	KC333050.1		

Table S3. List of primers used for vector plasmid construction and recombinant virus characterization.

Primer	Sequence (5'-3')
NP-5′UTR-F	GTTAAGAAAAAAGTACACTTGGGGGCGACAACGCCTGATCTCGACCCCGATACCGAGACACAGGATTTGTTGCTTGTATCCTTTCCTCTTTCCACCGAGATAATCATGGAGACAGTATCACTAAT
NP-5′UTR-R	ATTAGTGATACTGTCTCCATGATTATCTCGGTGGAAAGAGGAAAGGATACAAGCAACAAATCCTGTGTCTCGGTATCGGGGTCGAGATCAGGCGTTGTCGCCCCCAAGTGTACTTTTTTCTTAAC
NP-3′UTR-F	GATGCAACATTTGTATATAAATCGCTGAGCAGATCACGCACAGCCACCCTCTAATTGATCGCAACCTGGACAACACATTCGAAATGGTAATTAAATATTAAGAAAAAATGAATTTGGGGgcgAATGCCTCAAAAT
NP-3′UTR-R	ATTTTGAGGCATTcgcCCCCAAATTCATTTTTTCTTAATATTTAATTACCATTTCGAATGTGTTGTCCAGGTTGCGATCAATTAGAGGGTGGCTGTGCGTGATCTGCTCAGCGATTTATATACAAATGTTGCATC
P-5′UTR-F	GTTAAGAAAAAAGTACACTTGGGGGCGAATCCCACTCAAGTCAGGTGGCGCCAGTTTCAGCCCTGACAACCGCACACGACACCAGCTAGACATGGAGACAGTATCACTAAT
P-5′UTR-R	ATTAGTGATACTGTCTCCATGTCTAGCTGGTGTCGTGTGCGGTTGTCAGGGCTGAAACTGGCGCCACCTGACTTGAGTGGGATTCGCCCCCAAGTGTACTTTTTTCTTAAC
P-3′UTR-F	GATGCAACATTTGTATATAAGGCAAGCAGCATCAACAGCCACTCAGTCAGACCAGCCCGAGCATCCAGATCTCCACACCTGTAGGGCCCTAGACAAACAAGCTGTCCATGTTAAGAAAAAAGTACACTTGGGGgcgAATGCCTCAAAAT
P-3′UTR-R	ATTTTGAGGCATTcgcCCCCAAGTGTACTTTTTTCTTAACATGGACAGCTTGTTTGTCTAGGGCCCTACAGGTGTGGAGATCTGGATGCTCGGGCTGGTCTGACTGAGTGGCTGTTGATGCTGCTTGCCTTATATACAAATGTTGCATC
M-5′UTR-F	GTTAAGAAAAAAGTACACTTGGGGgcgAATGCCTCAAAATCCTGACCTACCCTAAGCATTCCGCAGCCATGGAGACAGTATCACTAAT
M-5′UTR-R	ATTAGTGATACTGTCTCCATGGCTGCGGAATGCTTAGGGTAGGTCAGGATTTTGAGGCATTcgcCCCCAAGTGTACTTTTTTCTTAAC
M-3′UTR-F	GATGCAACATTTGTATATAATCGCAAGATGATTATACAGTTAAGAAAAAATAACAAGAGCTATCATTAAGTGTGGGGgcgAATGCCTCAAAAT
M-3′UTR-R	ATTTTGAGGCATTcgcCCCCACACTTAATGATAGCTCTTGTTATTTTTTCTTAACTGTATAATCATCTTGCGATTATATACAAATGTTGCATC
F-5′UTR-F	GTTAAGAAAAAAGTACACTTGGGGGCGAATGCCCATAATAAACCGACTCGACAGAACTCACCATACTGTGGCAACCAGACATGGAGACAGTATCACTAAT
F-5′UTR-R	ATTAGTGATACTGTCTCCATGTCTGGTTGCCACAGTATGGTGAGTTCTGTCGAGTCGGTTTATTATGGGCATTCGCCCCCAAGTGTACTTTTTTCTTAAC
F-3′UTR-F	GATGCAACATTTGTATATAACAACGCCCGTGGGTCTGGGTCTCCCACAGTGCACCAAGGGACCGGCACAGTCAACACGAATGCAGTGCTACAGCATTCCGCTAATTAAGAAAAAATTGTGGGGgcgAATGCCTCAAAAT

F-5′UTR-R	ATTTTGAGGCATTcgcCCCCACAATTTTTCTTAATTAGCGGAATGCTG TAGCACTGCATTCGTGTTGACTGTGCCGGTCCCTTGGTGCACTGTGGG AGACCCAGACCCACGGCGTTGTTATATACAAATGTTGCATC
HN-5′UTR-F	GTTAAGAAAAAAGTACACTTGGGGGCGAATGCCcATTTGCAGTGATC ATTCAATTGTCCTAGTTGTACTAAACTGAGTATCTTTCTCCCAATGGA GACAGTATCACTAAT
HN-5′UTR-R	ATTAGTGATACTGTCTCCATTGGGAGAAAGATACTCAGTTTAGTACA ACTAGGACAATTGAATGATCACTGCAAATgGGCATTCGCCCCCAAGT GTACTTTTTTCTTAAC
HN-3′UTR-F	GATGCAACATTTGTATATAACGGCCCTGTTGGCTGTGCCATGAAGCC AAAAGTAGACTCATATTATCTCTTGTCTATATGCAGTTTAATAAAAA ACCAAATGTGGGGgcgAATGCCTCAAAAT
HN-3′UTR-R	ATTTTGAGGCATTcgcCCCCACATTTGGTTTTTATTAAACTGCATATA GACAAGAGATAATATGAGTCTACTTTTGGCTTCATGGCACAGCCAAC AGGGCCGTTATATACAAATGTTGCATC
L-5′UTR-F	GTTAAGAAAAAAGTACACTTGGGGGCGAATGCCCGATCACCATGGA GACAGTATCACTAAT
L-5′UTR-R	ATTAGTGATACTGTCTCCATGGTGATCGGGCATTCGCCCCCAAGTGT ACTTTTTTCTTAAC
L-3′UTR-F	GATGCAACATTTGTATATAAGATTCGTGGAGTCTGTCTAACACACAG TCGAGTCGTTGTGTACACCGCAATGATTCTAACGGCACTTGGAGAAT GTGCAGTACACACCTGACATGGGAAAGTTTAAGAAAAAAAGTACAC TTGGGGgcgAATGCCTCAAAAT
L-3′UTR-R	ATTTTGAGGCATTcgcCCCCAAGTGTACTTTTTTTCTTAAACTTTCCCAT GTCAGGTGTGTACTGCACATTCTCCAAGTGCCGTTAGAATCATTGCG GTGTACACAACGACTCGACTGTGTGTTAGACAGACTCCACGAATCTT ATATACAAATGTTGCATC
nonUTR-HA-F	TTAAGAAAAAAGTACACTTGGGGGCGAATATGGAGACAGTATCAC
nonUTR-HA-F	GGCGCTTTTGAGGCATTcgcCCCCATTTTTTCTTAATTATATACAAATG TTGC
HA-F	ATGGAGACAGTATCACTAATAACTATACTAGTAGTAGC
HA-R	TTATATACAAATGTTGCATCTGCAAGACCC
T4-HA-F	AAGAGGCAGATCTATAATGC
T4-HA-R	GCTTGACTGTGGTCTGAGCC

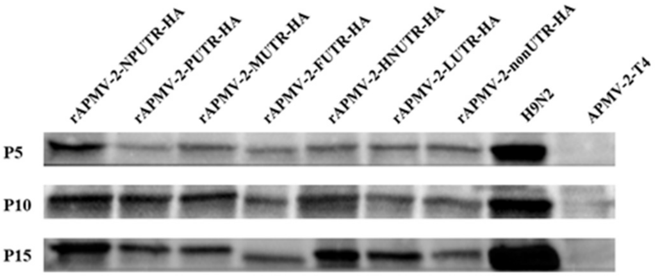

Figure S1: Stability analysis of APMV-2/HAs. APMV/HAs was grown sequentially in embryonated chicken eggs for 15 passages. CEF were infected with rAPMV-2/HAs at passage 5, 10 and 15. APMV-2-T4 and H9N2 were used as negative and positive control respectively. The expression of HA protein was evaluated by western blot analysis.
